# Successful treatment of multi-hit *TP53-*mutated myelodysplastic syndromes with erythroid predominance using allogeneic stem cell transplantation and ruxolitinib

**DOI:** 10.1007/s00277-025-06383-1

**Published:** 2025-05-06

**Authors:** Seigi Oshima, Junya Kanda, June Takeda, Takashi Sakamoto, Chisaki Mizumoto, Kouhei Yamashita, Yasuhito Nannya, Seishi Ogawa, Akifumi Takaori-Kondo

**Affiliations:** 1 Department of Hematology, Graduate School of Medicine, Kyoto University, Kyoto, Japan; 2 Division of Cancer Evolution, National Cancer Center Research Institute, Tokyo, Japan; 3 Department of Pathology and Tumor Biology, Graduate School of Medicine, Kyoto University, Kyoto, Japan; 4 Division of Hematopoietic Disease Control, Institute of Medical Science, The University of Tokyo, Tokyo, Japan

**Keywords:** Myelodysplastic syndromes, Acute erythroid leukemia, Multi-hit *TP53* mutation, Complex karyotype, Allogeneic stem cell transplantation, Ruxolitinib

## Abstract

*TP53*-mutated myelodysplastic syndrome (MDS) and acute erythroid leukemia (AEL) with complex karyotype have a very poor prognosis. The upregulation of the JAK-STAT pathway has been implicated in their pathogenesis, and inhibition of this pathway has shown promising disease control in preclinical models. Here, we report a case of refractory multi-hit *TP53*-mutated MDS with erythroid predominance on the verge of transitioning to AEL, which achieved hematological complete remission following allogeneic stem cell transplantation and ruxolitinib initiation. The patient exhibited chemoresistance to multiple regimens, including cytarabine with daunorubicin, high-dose cytarabine, and venetoclax with azacitidine. Despite the presence of residual disease post-transplant, complete remission was achieved two months after ruxolitinib initiation and tacrolimus tapering. At the 8-month follow-up, remission persists without evidence of relapse. This case highlights the potential of combining graft-versus-leukemia effects with ruxolitinib as a therapeutic strategy for *TP53*-mutated MDS/AEL. Further studies are warranted to evaluate the efficacy and safety of this strategy in a broader clinical setting.

## Introduction

Acute erythroid leukemia (AEL) is a rare type of acute myeloid leukemia, characterized by the proliferation of erythroid precursors in the bone marrow and peripheral blood. Currently, it is defined by the World Health Organization (WHO) 2022 criteria as ≥ 30% proerythroblasts and erythroid precursors constituting ≥ 80% of cellularity in bone marrow biopsy [1]. The International Consensus Classification describes this condition as acute myeloid leukemia with mutated *TP53*, with the presence of at least 20% of blasts or meeting the criteria for pure erythroid leukemia in the blood or bone marrow accompanied by a somatic *TP53* mutation with a variant allele frequency greater than 10% [2]. The frequent revision of the AEL definition has reclassified a subtype of AEL as myelodysplastic syndrome (MDS) based on the percentage of myeloblasts [3]. However, several studies have revealed that cytogenetic risk is a more important prognostic factor than the percentage of myeloblasts [4, 5]. MDS with erythroid predominance can be considered the same disease entity as AEL [5, 6].

MDS with erythroid predominance/AEL shows chemoresistance and dismal prognosis, with a median overall survival of 3–9 months from initial diagnosis [5, 7]; another report revealed that the median overall survival for adults was only 5 months, with a 1-year survival rate of 26.7% [8]. Allogeneic stem cell transplantation (allo-SCT) is the only curative therapy available for patients with relapsed and refractory acute myeloid leukemia and offers a chance for long-term remission. However, even with allo-SCT, the relapse rate of AEL is high, underscoring the need for novel therapeutic strategies. Recent studies have shown that AEL can be categorized into several groups based on genomic and molecular characteristics [9, 10]. *TP53* mutations are found in approximately 39% of patients with AEL, making it a unique subtype [9]. In *TP53*-mutated AEL, gain and focal amplification of *EPOR* and JAK/STAT pathway genes are frequently observed, suggesting that JAK2 inhibition may be a therapeutic option. Takeda et al. [9] confirmed the therapeutic potential of the JAK2 inhibitor ruxolitinib in an in vivo assay using a patient-derived xenograft model. These promising preclinical data suggest that targeting the JAK/STAT pathway may be a novel therapeutic avenue for patients with *TP53*-mutated AEL.

Here, we present an adult patient with refractory MDS with erythroid predominance and multi-hit *TP53* mutations, who was successfully treated with allo-SCT and ruxolitinib therapy.

## Case presentation

**Fig. 1:**
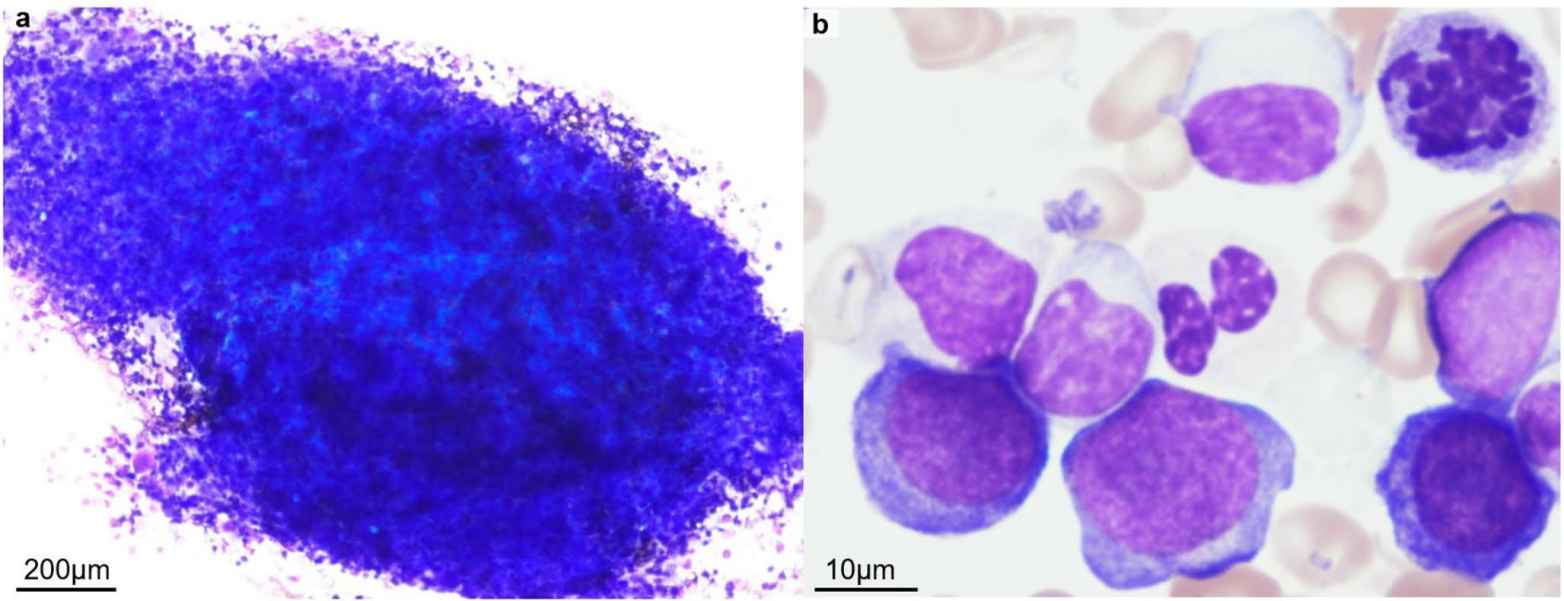
Morphological features of bone marrow aspirate on first presentation. Giemsa staining of the bone marrow samples (**a**) Severely hypercellular marrow with marked erythroid hyperplasia (**b**) Dysplastic precursor erythroid lineage showing irregular nuclear contours and coarse chromatin. The scale bar is estimated based on the average diameter of red blood cells (7.5 μm)

A healthy 50-year-old man presented to a local hospital with a fever and sore throat. Laboratory findings revealed anemia and mild leukocytosis with 3.0% blasts, and the patient was referred to our hospital. At presentation, laboratory findings were a hemoglobin level of 8.0 g/dL; platelet count of 394,000/µL; and white blood cell count of 11,580/µL with 45% neutrophils, 14% lymphocytes, 1% eosinophils, 2% metamyelocytes, and 38% myelocytes, with blasts outside count; and an elevated lactate dehydrogenase level of 258 U/L (reference range: 124–222 U/L). Bone marrow examination revealed severe hypercellular marrow with trilineage dysplasia and erythroid hyperplasia (Fig. 1).

Myeloblasts were within normal limits (2.0%), whereas proerythroblasts were elevated (9.6%). The ratio of myeloid cells to erythroid cells was 0.68, indicating erythroid hyperplasia. Immunophenotyping identified two abnormal cell populations: (1) 2.5% of cells positive for CD7, CD13, CD33, CD34, CD117, HLA-DR, and CD38 with partial expression of CD4, and (2) 9.1% of cells positive for CD36, CD71, and CD117 with partial expression of CD7 and HLA-DR, which presumably corresponded to myeloblasts and proerythroblasts, respectively. A bone marrow biopsy also demonstrated mild myelofibrosis and hypercellular marrow (95%) with increased blasts and erythroid lineage (> 80%).

**Fig. 2:**
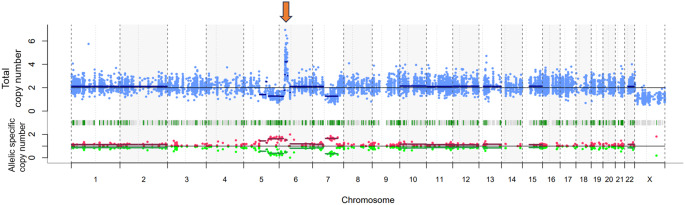
Genome-wide copy number and allele-specific copy number analysis. Copy number profile generated from next-generation sequencing data demonstrates multiple chromosomal aberrations consistent with a complex karyotype, including deletion of 5q, -7, and amplification chromosome 6p. The blue dots in the top panel indicate the total copy number, while the red and green dots in the bottom panel represent allelic specific copy number. A focal high-level amplification on chromosome 6p, including the *PIM1* locus, is indicated by the orange arrow

Further cytogenetic analysis revealed a complex karyotype with del (5q), -7, and add (6) (p11.2). Next-generation sequencing (NGS) identified *TP53* p.R249G with a variant allele frequency (VAF) of 75% and *DNMT3A* p.R882C along with amplification 6p including the *PIM1* locus (Fig. 2).

Although deletion or loss of heterozygosity of the *TP53* locus could not be confirmed on NGS, the high VAF and the presence of a complex karyotype fulfilled the criteria for multi-hit *TP53* as defined in the 2022 International Consensus Classification [2]. Thus, a diagnosis of MDS with multi-hit *TP53* alteration (Revised International Prognosis Scoring system 6.5 points) was made [2, 11]. Given the significant increase in erythroid precursor cells (> 80%), the disease condition was considered the stage almost transitioning to AEL. Based on the diagnosis of *TP53*-mutated MDS with increased blast and erythroid predominance, the use of venetoclax with azacitidine (AZA: 70 mg/m²) was initiated. This treatment was chosen because of the known chemorefractory nature of *TP53*-mutated myeloid malignancies, favoring a less intensive approach. Additionally, CPX-351 was not yet approved in Japan at that time. VEN was administered in combination with an azole-class antifungal agent, with a dose escalation from 50 mg on day 1, 100 mg on day 2, and 200 mg daily from day 3. VEN was discontinued on day 14 when the patient developed cellulitis. Two weeks later, a follow-up bone marrow examination revealed a further increase in erythroid precursor cells, consistent with induction failure. We then changed the treatment to daunorubicin with cytarabine (DNR/AraC) (DNR: 50 mg/m², AraC: 100 mg/m²), which was ineffective. High-dose cytarabine (3 g/m² for 2 days) was administered to reduce the tumor burden. However, severe hypercellularity persisted with a gradual increase in the number of blasts and erythroid precursors.

**Fig. 3:**
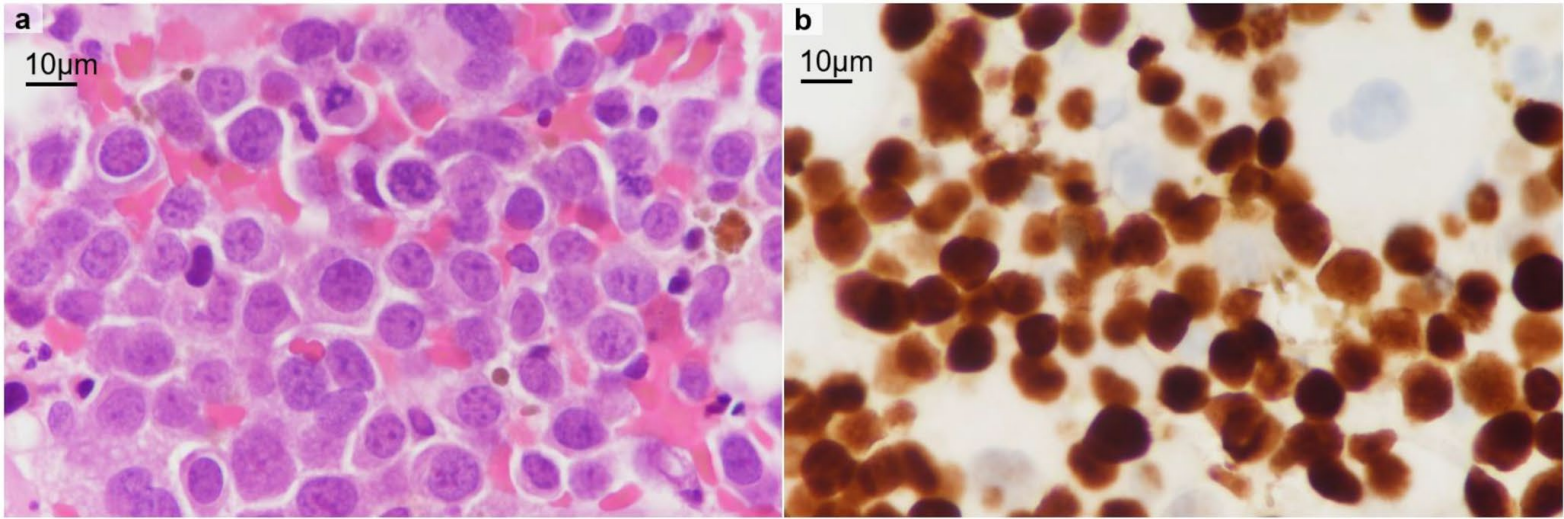
Histopathological findings of bone marrow one month after the allogeneic stem cell transplantation. (**a**) Hematoxylin and eosin (H&E) staining of the bone marrow biopsy specimen showing clusters of blasts with high nuclear-to-cytoplasmic ratios and prominent nucleoli. (**b**) Immunohistochemical staining for p53 revealing nuclear accumulation, indicative of *TP53*-mutated leukemia clones. The scale bar is estimated based on the average diameter of red blood cells (7.5 μm)

Given the refractory nature of the disease, allo-SCT was performed as salvage therapy to achieve disease control. Three months after the first presentation to our hospital, we performed unrelated peripheral blood stem cell transplantation from an HLA full-matched donor. The conditioning regimen consisted of fludarabine (150 mg/m²), melphalan (140 mg/m²), and total body irradiation (6 Gy). Graft-versus-host disease (GvHD) prophylaxis included tacrolimus and short-term methotrexate administration. The transplantation course was favorable except for the development of acute GvHD (stage 1) in the skin, which was successfully treated with topical steroids. A bone marrow examination performed on day 29 after transplantation revealed dysplasia in the erythroid lineage cells and 0.9% CD36-, CD71-, and CD117-positive leukemia cells. Post-transplantation chimerism analysis using fluorescence in situ hybridization (FISH) for sex chromosomes revealed that 5% of the bone marrow cells were recipient-derived. Pathological images showed clustering of p53-mutant blast cells in 20% of the examined bone marrow (Fig. 3). This discrepancy can be explained by focal expansion of residual recipient-derived leukemic clones. These findings indicated the persistence of leukemia cells.

**Fig. 4:**
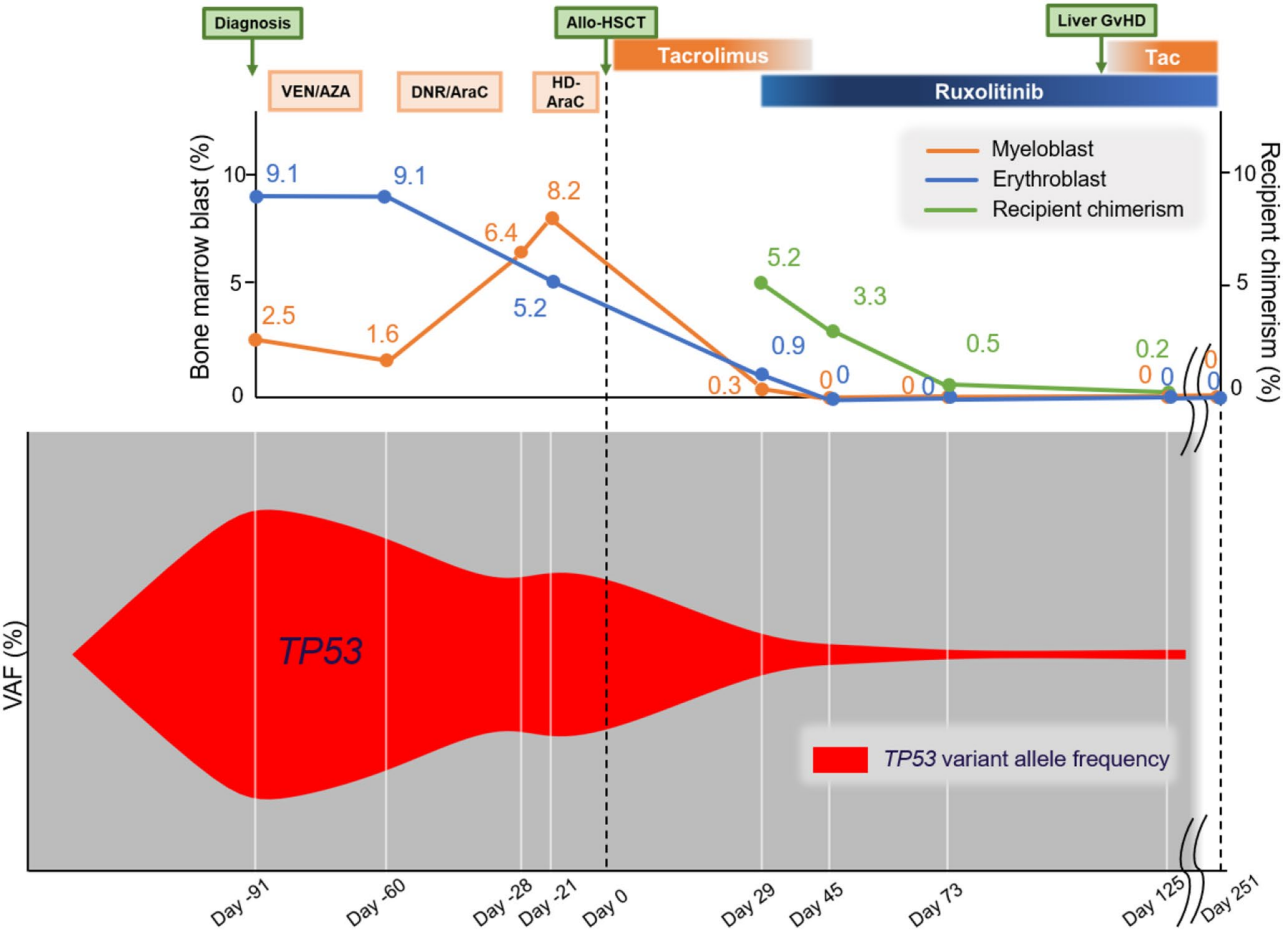
Dynamics of bone marrow blasts, recipient chimerism, and clonal evolution during the treatment course. The upper panel shows the percentages of bone marrow myeloblasts, erythroblasts, and recipient chimerism during the treatment course. Based on the next-generation sequencing results, the lower panel displays a fish plot illustrating the clonal evolution of *TP53*-mutated clone (red). The clone size represents the relative variant allele frequency. *Abbreviations*: VEN/AZA: venetoclax and azacitidine, DNR/AraC: daunorubicin and cytarabine, HD-AraC: high-dose cytarabine, Allo-HSCT: allogeneic hematopoietic stem cell transplantation, Tac: tacrolimus

To enhance the graft-versus-leukemia (GVL) effects, tacrolimus was rapidly tapered and discontinued within three weeks (Fig. 4). Simultaneously, we added the JAK2 inhibitor ruxolitinib (20 mg/day) to the treatment regimen with the primary aim of augmenting anti-leukemic activity. This decision was supported by previous reports suggesting a critical role of the EPOR/JAK-STAT pathway in AEL pathophysiology and suggested the therapeutic potential of targeting it [9]. Two weeks later, the ruxolitinib dose was increased to 40 mg/day; however, owing to the progression of cytopenia, the dose was decreased to 20 mg/day. Two months after tacrolimus discontinuation, chronic oral and liver GvHD developed, leading to tacrolimus re-initiation. However, bone marrow examination detected no leukemia cells with flow cytometry and p53 staining in the pathological specimen, consistent with complete hematological remission.

The transition in the clone size of the mutations is depicted based on the VAF data obtained from NGS (Fig. 4). Initially, *TP53*-mutated clones were dominant. This clone persisted after VEN/AZA therapy. Following DNR/AraC therapy, myeloblasts increased to 6.4%. *TP53* VAF rather expanded even after HD-AraC therapy, indicating its refractory nature and aggressiveness. Combining allo-HSCT, rapid tacrolimus tapering, and ruxolitinib therapy eradicated nearly all clones. These results suggest that the combined effect of GVL and ruxolitinib is a potential therapy for refractory *TP53*-mutated AEL. For oral and liver GvHD, 0.25 mg/kg of prednisolone was initiated and tapered off within two months, while maintaining the trough concentration of tacrolimus at 3–5 ng/mL. The patient remains in complete remission with no leukemia cells on flow cytometry detected at the 8-month post-transplant follow-up.

## Discussion

MDS/AEL, particularly the multi-hit *TP53*-mutated subtype, poses significant therapeutic challenges owing to its association with chemoresistance and poor outcomes. This is the first case report demonstrating the efficacy of combination therapy with allo-SCT and ruxolitinib in multi-hit *TP53*mutated MDS/AEL with a complex karyotype. Two explanations exist for the patient’s favorable response.

First, *TP53*-mutated AEL is frequently associated with hyperactivation of the EPOR/JAK/STAT pathway, driven by gains or amplifications in these signaling genes. Preclinical studies, including those by Takeda et al. [9], have demonstrated that ruxolitinib, a selective JAK2 inhibitor, suppresses tumor growth and prolongs survival in xenograft models of *TP53-*mutated AEL. NGS revealed chromosome 6p amplification, including at the *PIM1* locus. *PIM1* has been identified as a downstream effector of the JAK/STAT pathway; STAT proteins bind to the *PIM1* promoter to induce its expression [12]. Ruxolitinib may directly contribute to tumor suppression by inhibiting this highly elevated pathway.

Second, ruxolitinib may have maintained GVL effects while suppressing GvHD caused by the rapid tapering of tacrolimus. One of the primary concerns associated with rapid immunosuppressant tapering is the increased risk of GvHD. In one study, rapid cyclosporine tapering resulted in grade III/IV acute GVHD in 27% of patients, with one fatality [13]. However, after tacrolimus tapering in our study, the patient did not develop GVHD in the two months before achieving complete hematological remission. Ruxolitinib has been shown to improve outcomes in steroid-refractory GVHD while maintaining GVL effects [14]. Previous studies have shown that ruxolitinib enhances GVL effects by modulating immune cell function without impairing the ability of the donor immune system to target leukemia cells [15, 16]. In this case, the concurrent use of ruxolitinib during tacrolimus tapering may have prevented severe GVHD while maintaining the GVL effect.

The synergistic effects of these mechanisms may be critical in overcoming the aggressive nature of *TP53*-mutated MDS/AEL. This report highlights the therapeutic potential of combining the effects of GVL and ruxolitinib in the management of multi-hit *TP53*-mutated MDS/AEL. This approach offers a promising option for addressing the chemoresistance and high relapse rates in patients with MDS/AEL. Further studies are needed to evaluate the efficacy and safety of this strategy in a broader clinical setting.

## Data Availability

No datasets were generated or analysed during the current study.
